# ADHD symptoms among school-age children in Monastir (Tunisia): A cross-sectional study

**DOI:** 10.1192/j.eurpsy.2025.1137

**Published:** 2025-08-26

**Authors:** A. Guedria, N. Maatallah, R. Ayoub, A. Beji, T. Brahim, H. Ben Abid

**Affiliations:** 1 Child and adolescent Psychiatry, Fattouma Bourguiba university Hospital- University of Monastir, Monastir, Tunisia

## Abstract

**Introduction:**

Attention deficit disorder with or without hyperactivity (ADHD) is a neurodevelopmental disorder. It represents the most common psychiatric disorder in pediatric population. Children with ADHD can experience academic and social difficulties, as well as psychological complications.

**Objectives:**

The objective was to determine the prevalence rate and the clinical profile of children with ADHD symptoms in the governorate of Monastir (Tunisia) and to study the comorbid symptoms.

**Methods:**

We carried out a cross-sectional study, applying the Strengths and Difficulties Questionnaire (SDQ) scale and the short versions of Conners 3 to parents and teachers of 435 school children in a sample of 18 public and private schools randomly selected from 6 delegations in the governorate of Monastir.

**Results:**

The prevalence of ADHD symptoms was 12% of which 51.9% were boys and 48.1% were girls. The average age was 9.8 [9.2;10.4] years.

We found a predominance of the inattentive form with a frequency of 57.7% compared to the impulsive-hyperactive and combined forms which have a frequency of 17.3% and 25% respectively.

In 40% of the ADHD group, an emotional disorder was found, and in 54% of cases there were behavioral disorders, with a statistically significant difference. Relationship problems rise to 74%, but with no statistically significant difference.

**Conclusions:**

The prevalence of ADHD symptoms in the governorate of Monastir is similar to the rate found in other parts of the world. Identifying predisposing factors helps early intervention, which in turn reduces the psychosocial impact of this disorder.

**Disclosure of Interest:**

None Declared

